# De novo *SETD5* missense variants in individuals with MRD23 cluster within the protein’s SET domain

**DOI:** 10.1007/s00439-026-02882-x

**Published:** 2026-11-05

**Authors:** Daphna Marom, Yvonne Hilhorst-Hofstee, Sarina G. Kant, Mira Kharbanda, Fiona Osborne, Lisa Robertson, Noa Zunz Henig, Adi Mory, Omri Weiss, Deciphering Developmental Disorders (DDD) Study, Uri Hamiel, Emil Elhanan, Yael Wilnai, Veronika Chernuha, Hagit Baris Feldman, Alina Kurolap

**Affiliations:** 1 The Genetics Institute and Genomics Center, Tel Aviv Sourasky University Medical Center, Tel Aviv, Israel; 2 Gray Faculty of Medical and Health Sciences, Tel Aviv University, Tel Aviv, Israel; 3 Department of Clinical Genetics, Leiden University Medical Center, Leiden, The Netherlands; 4 Department of Neuroscience, Erasmus University Medical Centre, Rotterdam, The Netherlands; 5 Wessex Clinical Genetics Service, University Hospital Southampton NHS Foundation Trust, Southampton, UK; 6 North of Scotland Genetics Service, Aberdeen Royal Infirmary, Aberdeen, UK; 7 East of Scotland Regional Genetics Service, Ninewells Hospital, Dundee, UK; 8 Metabolic Genetics Clinic, Dana-Dwek Children’s Hospital, Tel Aviv Sourasky University Medical Center, Tel Aviv, Israel; 9 Pediatric Neurology Institute, Dana-Dwek Children’s Hospital, Tel Aviv Sourasky University Medical Center, Tel Aviv, Israel

## Abstract

*SETD5* heterozygous loss-of-function (LoF) variants and 3p25.3 deletions containing the *SETD5* gene cause intellectual developmental disorder, autosomal dominant 23 (MRD23). We report five patients with an MRD23 phenotype caused by *SETD5* missense variants. We studied patients with de novo* SETD5* missense variants. A comprehensive literature search was conducted to reveal additional reports of *SETD5* missense and LoF variants. Phenotypes and protein localization of missense variants were reviewed and compared to those reported for *SETD5* LoF variants and 3p25.3 deletions. Computational analysis of the potential effect of *SETD5* missense variants was performed. Six patients with de novo* SETD5* missense variants were identified across five centers, gathered through the Deciphering Developmental Disorders (DDD) study database; four additional individuals with missense variants were reported in the DDD database. A comprehensive literature search revealed 12 unique individuals with missense variants, and 104 individuals with LoF variants or 3p25.3 deletions. One of the variants, p.Arg348Gln, recurred independently in three unrelated individuals. All patients with likely pathogenic missense variants shared dysmorphic features, GDD and autism, consistent with MRD23, but no major congenital anomalies, apart from hydrocephalus, which was a novel feature observed. Heart defects were reported only in the LoF group. Unlike LoF variants, which are scattered throughout the entire *SETD5* coding region, pathogenic missense variants were confined to the protein's SET domain. We show that disease-causing missense variants in *SETD5* cluster at the protein’s SET domain. This expands the mutation spectrum of the *SETD5*-related neurodevelopmental phenotype, providing a framework for improving both postnatal and prenatal genetic diagnostics.

**Supplementary Information:**

The online version contains supplementary material available at https://doi.org/10.1007/s00439-026-02882-x.

## Introduction

Heterozygous loss-of-function (LoF) variants in *SETD5* and 3p25.3 deletions containing the *SETD5* gene cause intellectual developmental disorder, autosomal dominant 23 (MRD23, OMIM #615761). MRD23 is characterized by variable degrees of intellectual disability (ID), with or without autism spectrum disorder, behavioral difficulties, learning difficulties and language delays, dysmorphism, variable congenital heart defects and polydactyly. Other reported manifestations include stuttering, brachycephaly, feeding difficulties, skeletal findings, gastrointestinal or genitourinary malformations, and epilepsy, including refractory focal epilepsy (Grozeva et al. 2014; Kuechler et al. 2015; Ahsan et al. 2023; Manokaran et al. 2023; Crippa et al. 2020; Callahan et al. 2025). Moyamoya disease has been described in two patients (Pinard et al. 2020; Dams et al. 2026). Recently, pigmentary retinopathy and central hypothyroidism were described in a 2-year-old girl, and are thought to be associated with a *SETD5* LoF variant (c.896_897del; p.Ile299Argfs*13) (Ahsan et al. 2023). A phenotypic overlap with KBG, Cornelia de Lange and Noonan syndromes have also been described (Crippa et al. 2020; Kim et al. 2025; Ansari et al. 2025). The vast majority of LoF *SETD5* variants linked to a clinical neurodevelopmental phenotype were de novo. Only two familial cases have been reported (Szczałuba et al. 2016; Powis et al. 2018). Interestingly, Powis et al. described three affected children from two unrelated families with maternal inheritance of LoF *SETD5* variants; in both families the mothers had normal intelligence, suggesting reduced penetrance (Powis et al. 2018).

To date, missense variants in *SETD5* have not been clearly implicated in a neurodevelopmental phenotype. We detected two children with de novo missense variants in the *SETD5* gene. Through collaboration via the Deciphering Developmental Disorders (DDD) study database (Firth and Wright 2011; Wright et al. 2023), four additional patients were identified, including one recurring variant. We suggest that *SETD5* missense variants that localize to the protein’s SET domain are more likely to lead to an MRD23 phenotype.

## Methods

### Identification of patients with SETD5 missense variants

**Fig. 1:**
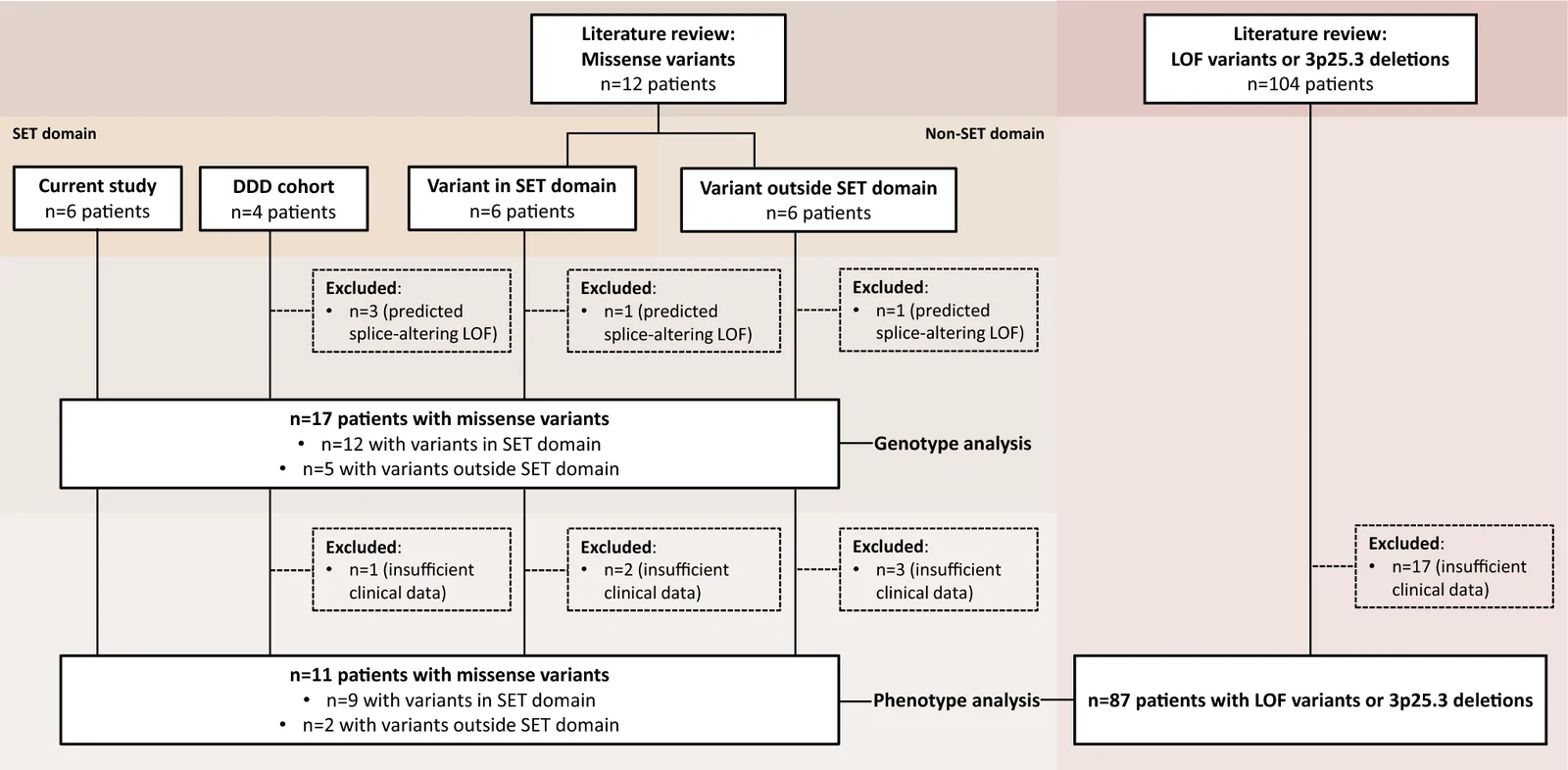
Flow chart describing the study outline and cohort assembly

Two patients (1 and 2; Table 1) were identified with de novo* SETD5* missense variants at Tel Aviv Sourasky University Medical Center, Israel, through diagnostic trio exome sequencing (ES) for evaluation of neurodevelopmental abnormalities and/or congenital anomalies. Since *SETD5* missense variants are not clearly recognized as disease-causing, we aimed to identify additional patients. For this, we explored the DDD study database ((https://www.deciphergenomics.org/ddd/overview), Firth and Wright 2011; Wright et al. 2023) and performed a comprehensive literature review by Google Scholar and PubMed mining using the search words: “SETD5”, “intellectual developmental disorder, autosomal dominant 23”, “mental retardation disorder, autosomal dominant 23”, “MRD23”, “OMIM 615761”, and “OMIM 615743”. Manuscripts reporting variants in this gene were initially included if the exact variant was mentioned, either in the main text or the supplementary material. We also manually screened the reference lists of all eligible publications to identify additional cases. Afterwards, the studies were examined for clinical data on an individual patient level, and only those with sufficient detail were used for the phenotype analyses. We excluded conference abstracts and review articles without specific patient data. See Fig. 1 for a flow chart of the study outline.

**Table 1:** Comparison of clinical features in patients with loss-of-function (LoF) and missense variants in SETD5

	LoF and 3p25.3del	Missense		
*n*	87	9		
*Inheritance*				
De novo	68 (out of 84, 81%)*	7 (out of 8, 88%)		
Inherited	13 (out of 84, 15%)*	1 (out of 8, 13%)		
*Clinical features*			Raw p-value	FDR-adjusted p-value
Dysmorphic features	75 (86%)	8 (89%)	1.000	1.000
GDD with or without ID	76 (87%)	8 (89%)	1.000	1.000
Autism	21 (24%)	7 (78%)	0.002	0.036
Neuropsychiatric and/or behavioral disorder	36 (41%)	5 (56%)	0.490	0.712
Stereotypic movements	9 (10%)	2 (22%)	0.274	0.469
Seizures	14 (16%)	3 (33%)	0.195	0.446
Hypotonia	38 (44%)	6 (67%)	0.293	0.469
Hydrocephalus	0	2 (22%)	0.008	0.051
Growth delay and/or short stature	30 (34%)	1 (11%)	0.2634	0.469
Cerebral vascular anomalies	0	1 (11%)	0.094	0.250
Congenital heart defect	27 (31%)	0	0.057	0.183
Dental anomalies	13 (15%)	5 (56%)	0.011	0.051
Polydactyly	10 (11%)	1 (11%)	1.000	1.000
Musculoskeletal abnormalities, other	29 (33%)	7 (78%)	0.013	0.051
Renal anomalies	7 (8%)	1 (11%)	0.559	0.745
Other	48 (55%)	6 (67%)	0.727	0.894

*Data on inheritance mode was available for 84 out of 87 individuals with LoF variants and 8 out of 9 individuals with missense variants

Phenotypic features of patients with missense variants were carefully reviewed, as available, and compared to those reported in the literature for patients with *SETD5* LoF variants and 3p25.3 deletions. Patient data from the literature were accounted in the analysis only if the phenotypes were sufficiently detailed in the publication. Variants were interpreted and classified according to the American College of Medical Genetics and Genomics and the Association for Molecular Pathology (ACMG-AMP) guidelines with modification according to the Clinical Genomics (ClinGen) sequence variant interpretation group. (Richards et al. 2015; Pejaver et al. 2022; Biesecker and Harrison 2018; ClinGen Sequence Variant Interpretation Group 2021, 2020; Durkie et al. 2024) De novo designation was applied only for variants identified via trio ES with confirmed parental status.

### Computational analysis of SETD5 missense variants

We performed computational analyses to study the pathogenicity and the potential effect of the identified missense variants on protein structure and function. First, variant pathogenicity was assessed using multiple pathogenicity assessment software (REVEL, CADD, AlphaMissense) and conservation was evaluated using GERP, ConSurf and Aminode. The abundance of missense variants across the *SETD5* gene was assessed in gnomAD v4.1, and regional missense constraint was assessed in gnomAD v2.1.1 (Chao et al. 2024).

Since disease-causing variants appeared to localize to the protein SET domain, we modelled the domain’s structure using SWISS-MODEL and AlphaFold; structure quality was assessed using the QMEAN v4.5.0 tool (https://swissmodel.expasy.org/qmean/) and the better quality model was chosen for subsequent analyses. The model was used to visualize the 3D localization of the affected residues within the domain, to test for potential effects they may exert on protein stability (by mCSM, SDM and DUET servers), and protein surface electrostatic potential analysis (using the Adaptive Poisson-Boltzmann Solver, APBS, software). The SET domain predicted structure and mutated residues were visualized using UCSF Chimera software.

### Statistical analysis

To compare the prevalence of clinical features between the LoF and missense variant cohorts, we employed the two-sided Fisher’s Exact Test. This test was selected as the standard for comparing proportions between two groups with small sample sizes. A *p-value* of < 0.05 was considered statistically significant. The Benjamini–Hochberg False Discovery Rate (FDR) was applied to account for multiple comparisons. Due to the small number of patients, this analysis was regarded as exploratory.

## Results

### Clinical and genetic findings in individuals carrying SETD5 missense variants

A total of 22 patients with missense SETD5 variants were identified (16 with variants in the SET domain and 6 outside the domain). Our cohort of missense variant carriers includes six unrelated patients (age range 2.5–19 years) harboring three *SETD5* (NM_001080517.3) heterozygous de novo missense variants: p.Ile387Thr (Patient 1), p.Arg348Gln (Patients 2–4), p.Gly344Asp (Patient 5) and p.Ser353Leu (Patient 6); the p.Arg348Gln variant recurred in three unrelated patients, one of whom had an Episign methylation profile consistent with the diagnosis of MRD23 with moderate confidence (Table S1). An additional likely pathogenic variant in *ZBTB7A* (c.624_625del; p.Val209Glyfs*40) was identified in Patient 2, suggesting potential double diagnosis contributing to the observed neurodevelopmental phenotype (Tables S1). Four additional individuals with variants affecting the SET domain: p.Asn345Tyr, p.Asn314Ser (two patients) and p.Tyr391Cys were reported in the DDD Study database with relevant phenotype HPO terms; however, detailed clinical data was not available. Two of these variants (p.Asn314Ser and p.Tyr391Cys) were predicted to affect mRNA splicing (SpliceAI scores 0.88 and 0.99, respectively), indicating that they are essentially LoF variants; therefore, they were removed from further analysis. Literature review identified an additional six missense variants located in the SET domain, one of which was p.Tyr391Cys (predicted to affect splicing), and only three of the remaining with sufficient clinical data reported (Pinard et al. 2020; De Falco et al. 2025; Odhams and Williams 2025; Abul-Husn et al. 2023; Sadikovic et al. 2021). Six missense variants located outside the SET domain were reported in the literature, and only two of these patients had sufficient clinical data); (Pinard et al. 2020; De Falco et al. 2025; Rivera-Munoz et al. 2025; Lin et al. 2022) of these variants, p.Ser655Arg, was also predicted to affect the gene’s splicing (SpliceAI 0.95), and was considered as an LoF. Overall, sufficient clinical data was available for 75% patients (n=9/12) with true missense variants located in the SET domain (De Falco et al. 2025; Odhams and Williams 2025). In addition, we identified 104 reported individuals with either LoF *SETD5* variants or 3p25.3 deletions, of which 87 (81%) had sufficient clinical data available in the publications to allow phenotype comparisons between LOF and missense *SETD5* variant type groups (Callahan et al. 2025; Kim et al. 2025; Pizzo et al. 2019; Yagasaki et al. 2018; Green et al. 2017; Stur et al. 2017; Pan et al. 2020; Fan et al. 2025; Luppino et al. 2025; Pande et al. 2024; Shangguan et al. 2024; Pires et al. 2020; Fang et al. 2019; Aoi et al. 2019).

The nine informative patients with missense variants in the SET domain (six from our cohort and three from the literature) shared dysmorphic features previously described for MRD23 (Table S1). Additional phenotypic features in patients with SET domain missense variants included: early childhood speech and motor delay (n = 8/9, 89%), hypotonia (n = 6/9, 67%), dental anomalies (n = 5/9, 56%), autism (n = 7/9, 78%) and musculoskeletal abnormalities not including polydactyly (n = 7/9, 78%). Hydrocephalus (n = 2/9, 22%) was a novel feature observed only in the missense variant cohort; although one of these patients has a second diagnosis (*ZBTB7A* LoF), this disorder is not characterized by hydrocephalus, and this feature was observed in another patient in our cohort, emphasizing its potential as a novel disease characteristic. Cerebral venous anomalies were diagnosed in one patient (11%) at the age of 5 years. GDD/ID was reported in 89% of patients (n = 8/9) with missense variants, but it was in the mild to moderate range in all five patients (56%) in our cohort. These frequencies, observed in a limited patient group, require confirmation in larger, systematically phenotyped cohorts.

Conversely, in patients with *SETD5* LoF variants, GDD and ID (n=76/87, 87%) were more commonly reported, while hypotonia (n = 38/87, 44%), autism (n = 21/87, 24%) and dental anomalies (n = 13/87, 15%) were less common than in our missense variant carriers. Heart defects were reported only in the LoF variants group (n = 27/87, 31%). Genotype–phenotype comparisons between groups revealed that only autism (*p* = 0.002), hydrocephalus (*p* = 0.008), dental anomalies (*p* = 0.011) and musculoskeletal anomalies (*p* = 0.013) reached statistical significance. However, after correction for multiple testing, only autism remained significant (*p* = 0.036), likely due to the small number of patients in the missense group, meaning these results should be interpreted with caution. Additional data are available in Tables 1 and S1.

Interestingly, during our manual curation, we identified a patient from the Genomics England renal and urinary tract disorders disease group, who had a missense variant (p.Arg348Leu) (Odhams and Williams 2025) at the same position as the recurring *SETD5* p.Arg348Gln variant in our cohort. Two variants: p.Asp339Tyr and p.Arg767Cys were reported in a Moyamoya vasculopathy pediatric cohort, but were not thought by the authors to be related to the phenotype (Pinard et al. 2020). The p.Phe324Leu variant was reported as detected through reanalysis of diagnostic next-generation sequencing in children for various clinical indications (Abul-Husn et al. 2023; Sadikovic et al. 2021), p.Arg77Cys was reported in a cohort of patients with obsessive compulsive disorder (OCD) (Lin et al. 2022), and p.Gly1045Ser was reported as part of a cohort of patients with congenital anomalies of kidney and urinary tract (CAKUT) (Rivera-Munoz et al. 2025), which also included two patients with LoF variants in *SETD5*.

### Deleterious SETD5 missense variants cluster in the SET domain

The SWISS-MODEL homology-based SET domain prediction used the crystal structure of SETD2 bound to H3K36M and SAH (PDB 5v22) as a template (Sessa et al. 2019). This structure received a QMEANDisCo Global score of 0.53 ± 0.07 and QMEAN4 Value of − 3.71. Since SETD5 has a non-canonical SET domain with only 24% homology with SETD2, (Sessa et al. 2019) we also tested the structure predicted by AlphaFold. This structure received a slightly better QMEANDisCo Global score (0.58 ± 0.06) and QMEAN4 Value (− 2.61); therefore, the AlphaFold model was used for subsequent analyses.

**Fig. 2:**
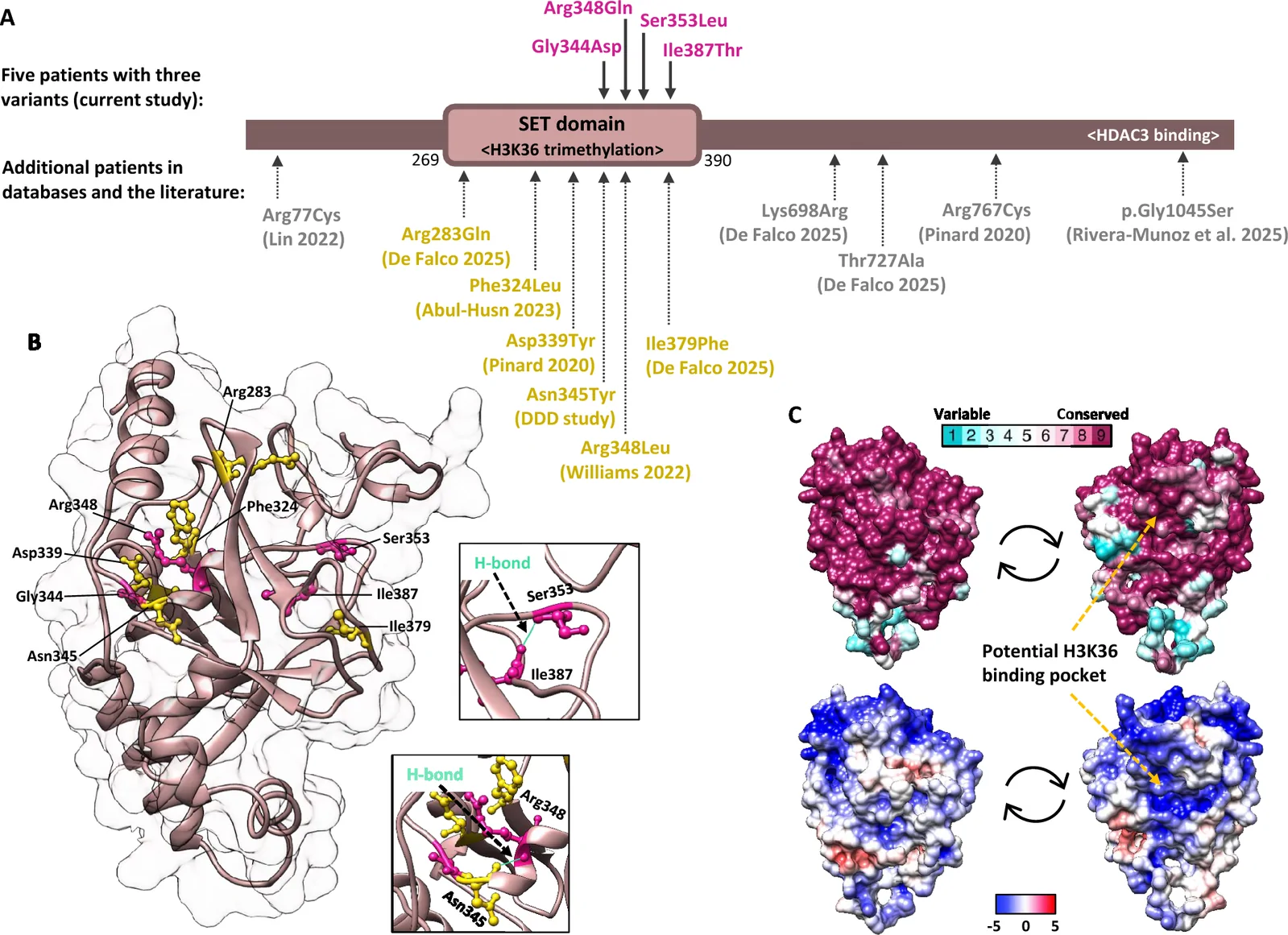
Disease-causing missense variants in SETD5 cluster in the SET domain. **A** Schematic representation of the SETD5 protein, showing clustering of variants to the SET domain (domain annotation according to UniProt Q9C0A6). **B** 3D structure of the SET domain bound to H3K36M (by similarity to SETD2, PDB: 5v22). Variants identified in this study (magenta) and previously reported (yellow) are shown to cluster in two parallel pockets. **C** Conservation analysis (by ConSurf, top) and surface electrostatics (APBS, buttom) of the SET domain 3D structure show that this domain contains two parallel pockets which are highly conserved and positively charged

**Fig. 3:**
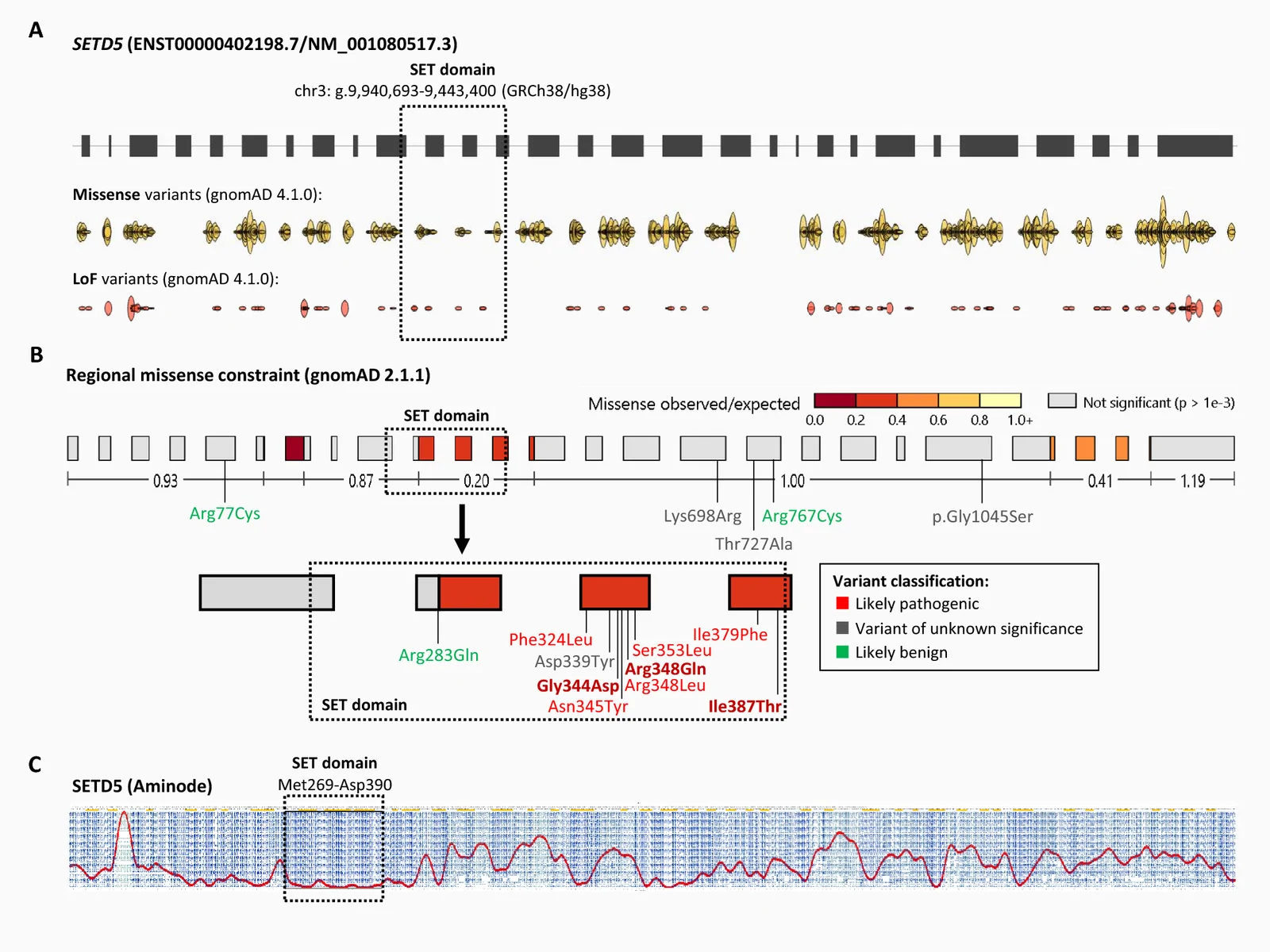
SETD5 region specific variant constraint and conservation. **A** gnomAD 4.1 representation of the SETD5 exons, showing the abundance of LoF and missense variants across the entire gene. While there is a paucity of LoF variants across the entire coding sequence (corresponding with pLI 1), missense variants are abundant throughout the gene, except for a clear paucity in the SET domain-coding region. **B** Regional missense constraint analysis available in gnomAD 2.1.1 shows clear constraint across the SET domain-coding region (Met269-Arg283 observed/expected: 0.8673, p = 0.3; Arg283-Asp390 observed/expected: 0.2029, p = 1.762–10). Missense variants are marked and colored by pathogenicity classification; variants observed in our cohort of patients are highlighted in bold. **C** Aminode amino acid conservation analysis reveals that the SET domain is the most conserved region of the protein

Disease-causing LoF variants are scattered throughout the entire coding region of the *SETD5* gene; however, structural analysis of the SETD5 protein revealed that missense variants that are presumed deleterious, as reported here, are confined to the protein's SET domain (Figs. 2, 3). Indeed, pathogenicity and protein stability assessments suggest detrimental effects of most of the variants reported in this domain on the SETD5 protein structure and its ability to bind other proteins, such as histones or nucleic acids. Structural analysis revealed two highly conserved and positively charged pockets located in parallel on each side of the SET domain 3D structure (Fig. 2C). Positively charged regions often form binding pockets for negatively charged ligands, nucleic acids, or interfaces for protein–protein interaction; one of these regions in SETD5 potentially functions in H3K36 histone binding (by similarity to SETD2, PDB: 5v22) (Sessa et al. 2019). Despite the low sequence homology between the SET domains of SETD5 and SETD2, it was previously shown that the critical residues required for its catalytic activity, SAM binding and substrate recognition, are highly conserved (Sessa et al. 2019). Importantly, all mutated residues classified as likely pathogenic appeared to be located inside these two parallel regions (Fig. 2B, C), highlighting their importance to proper protein function. Moreover, two of the amino acids mutated in our cohort (Ile387 and the recurrent Arg348) appeared to form a hydrogen bonds with other amino acids (Ser353 and Asn345, respectively), in which variants were observed in our cohort or reported in the DDD study database (Fig. 2B). Any variant affecting these residues is likely to disrupt hydrogen bond formation and abrogate 3D protein structure assembly. Interestingly, p.Ser353Leu was the only variant predicted to have a mild stabilizing effect on protein structure, while all other variants are predicted to destabilize it (Table 2). Further evidence supporting the pathogenicity of missense variants located in the SET domain can be observed by analyzing gnomAD data for this gene. While *SETD5* is clearly LoF intolerant (pLI = 1), the missense variant constraint is not as clear, with a Z score of 2.04 (gnomAD 4.1). Indeed, when assessing the abundance of LoF and missense variants across the gene, it is clearly noted that while LoF variants are scarce throughout the entire gene, missense variants appear to be relatively common (Fig. 3A). However, a clear paucity of missense variants can be observed within the exons encoding the SET domain (Fig. 3A). This is even further supported by the regional missense constraint data available in gnomAD 2.1.1, showing clear constraint in the SET domain encoding exons (Fig. 3B). Moreover, amino acid conservation analysis with Aminode shows that the SET domain is the most conserved region in the entire protein (Fig. 2C), and ConSurf reveals high conservation of the SET domain parallel pockets described above (Fig. 1C).

**Table 2:** Genetic characteristics of SETD5 missense variants located within the SET domain

SETD5 (NM_001080517.3) missense variants located within the SET domain										
Variant	c.1160T>C; p.Ile387Thr	c.1043G>A; p.Arg348Gln	c.1031G>A; p.Gly344Asp	c.1058C>T; p.Ser353Leu	c.1033A>T; p.Asn345Tyr	c.1015G>T; p.Asp339Tyr	c.972T>G; p.Phe324Leu	c.1043G>T; p.Arg348Leu	c.848G>A; p.Arg283Gln	c.1135A>T; p.Ile379Phe
Source	Patient 1 (Current)	Patients 2–4 (Current)	Patient 5 (Current)	Patient 6 (Current)	DDD study (Firth and Wright 2011)	Pinard et al. (2020)	Abul-Husn et al. (2023)	Odhams and Williams (2025)	De Falco et al. (2025)	De Falco et al. (2025)
Genomic location (hg19)	chr3-9485074 T>C	chr3-9483895 G>A	chr3-9483883 G>A	chr3-9483910 C>T	chr3-9483885 A>T	chr3-9483867 G>T	chr3-9483824 T>G	chr3-9483895 G>T	chr3-9483314 G>A	chr3-9485049 A>T
Genomic location (hg38)	chr3-9443390 T>C	chr3-9442211 G>A	chr3-9442199 G>A	chr3-9442226 C>T	chr3-9442201 A>T	chr3-9442183 G>T	chr3-9442140 T>G	chr3-9442211 G>T	chr3-9441630 G>A	chr3-9443365 A>T
Inheritance	de novo	de novo	de novo	NA	de novo	NA	de novo	de novo	Maternal	de novo
Exon	11	10	10	10	10	10	10	10	9	11
Protein domain	SET	SET	SET	SET	SET	SET	SET	SET	SET	SET
gnomAD MAF v2.1.1/v4.1	0/0	0/0	0/0	0/0	0/0	0/0	0/0	0/0	0.0016% (4 het)/0.0004% (7 het)	0/0
Franklin Community	0	0	0	0	0	0	0	0	3 het	1 het
phyloP	8.04	9.87	9.87	7.82	9.34	9.87	2.16	9.87	5.39	9.34
GERP	5.87	5.49	5.49	5.49	5.49	5.49	5.49	5.49	5.76	6.03
CADD	27.6	33	31	33	29	33	26.2	32	32	28.8
AlphaMissense	Deleterious (0.995)	Deleterious (0.998)	Deleterious (1.0)	Deleterious (0.998)	Deleterious (0.998)	Deleterious (0.999)	Deleterious (1.0)	Deleterious (0.998)	Deleterious (0.906)	Deleterious (0.968)
REVEL	Deleterious (0.94)	Deleterious (0.83)	Deleterious (0.88)	Deleterious (0.93)	Deleterious (0.92)	Deleterious (0.88)	Deleterious (0.73)	Deleterious (0.88)	Uncertain (0.55)	Deleterious (0.96)
DUET (ΔΔG kcal/mol)	Destabilizing (− 2.936)	Destabilizing (− 1.308)	Destabilizing (− 2.205)	Stabilizing (0.427)	Destabilizing (− 0.555)	Destabilizing (− 0.707)	Destabilizing (− 1.055)	Destabilizing (− 0.931)	Destabilizing (− 0.246)	Destabilizing (− 1.744)
SDM (ΔΔG kcal/mol)	Destabilizing (− 3.36)	Destabilizing (− 1.58)	Destabilizing (− 2.0)	Stabilizing (0.44)	Destabilizing (− 0.65)	Destabilizing (− 0.72)	Destabilizing (− 2.01)	Stabilizing (0.9)	Destabilizing (− 0.96)	Destabilizing (− 0.94)
mCSM (ΔΔG kcal/mol)	Highly destabilizing (− 2.616)	Destabilizing (− 1.308)	Highly destabilizing (− 2.06)	Stabilizing (0.087)	Destabilizing (− 0.347)	Destabilizing (− 0.57)	Destabilizing (− 0.908)	Destabilizing (− 1.413)	Destabilizing (− 0.192)	Destabilizing (− 1.61)
Criteria	PP3_strong, PM2_supporting, PM1, PM5_supporting, PS2_moderate,	PM5_supporting, PM1, PP3_moderate, PS2_moderate, PS4	PS2_moderate, PM2_supporting, PP3_moderate, PM1	PM1, PP3_strong, PM2_supporting	PS2_moderate, PM2_supporting, PP3_moderate, PM1	PM2_supporting, PM1, PP3_moderate	PM1, PS2_moderate, PM2_supporting, PP3	PS2_moderate, PM2_supporting, PM5_supporting, PP3_moderate, PM1,	PM1, BS2	PM1, PM2_supporting, PP3_strong, PS2_moderate
Classification	LP	P	LP	LP	LP	VUS	LP	LP	LB	LP

Among variants reported in the SET domain (n = 10), only two (20%) did not reach likely pathogenic classification using ACMG criteria. The p.Asp339Tyr variant, reported by Stur et al. (2017), Pinard et al. (2020) was classified as a variant of unknown significance (VUS), and the p.Arg283Gln variant, reported by Powis et al. (2018), De Falco et al. (2025) was classified as likely benign. The latter was reported in gnomAD (Table 2) and is located on the border between the constrained region (Arg283-Asp390 observed/expected: 0.2029, *p* = 1.762^–10^) and the non-constrained region (Met269-Arg283 observed/expected: 0.8673, *p* = 0.3) of the SET domain (Fig. 3B). Unlike most of the variants in the SET domain, all variants outside it (n = 5), were classified as either VUS or likely benign.

Additional bioinformatic data on all identified missense variants can be found in Tables 2 and 3.

**Table 3:** Genetic characteristics of SETD5 missense variants located outside the SET domain

SETD5 (NM_001080517.3) missense variants located outside the SET domain					
Variant	c.2299C>T; p.Arg767Cys	c.229C>T; p.Arg77Cys	c.3133G>A; p.Gly1045Ser	c.2093A>G; p.Lys698Arg	c.2179A>G; p.Thr727Ala
Source	Pinard et al. (2020)	Lin et al. (2022)	Rivera-Munoz et al. (2025)	De Falco et al. (2025)	De Falco et al. (2025)
Genomic location (hg19)	chr3-9490267 C>T	chr3-9476069 C>T	chr3-9512551 G>A	chr3-9489680 A>G	chr3-9490147 A>G
Genomic location (hg38)	chr3-9448583 C>T	chr3-9434385 C>T	chr3-9470867 G>A	chr3-9447996 A>G	chr3-9448463 A>G
Interitance	NA	NA	NA	de novo	de novo
Exon	16	5	19	15	16
Protein domain	–	–	–	–	–
gnomAD MAF v2.1.1/v4.1	0.0079% (22 het)/0.0154% (248 het)	0.0016% (4 het)/0.0008% (13 het)	0/0.00006% (1 het)	0/0	0.0004012% (1 het)/0.0007436% (12 het)
Franklin Community	11 het	7 het	0	1 het	1 het
phyloP	5.64	3.99	2.3	8.34	8.94
GERP	4.85	4.75	3.52	5.81	5.76
CADD	28.1	32	21.2	25.9	25.5
AlphaMissense	Uncertain (0.367)	Benign (0.2)	Benign (0.076)	Benign (0.317)	Deleterious (0.859)
REVEL	Deleterious (0.81)	Uncertain (0.35)	Benign (0.26)	Deleterious (0.82)	Deleterious (0.8)
DUET (ΔΔG kcal/mol)	Destabilizing (− 0.24)	Destabilizing (− 0.278)	Destabilizing (− 1.083)	Stabilizing (0.164)	Stabilizing (0.188)
SDM (ΔΔG kcal/mol)	Destabilizing (− 0.45)	Destabilizing (− 0.52)	Destabilizing (− 3.13)	Stabilizing (0.05)	Stabilizing (0.32)
mCSM (ΔΔG kcal/mol)	Destabilizing (− 0.18)	Destabilizing (− 0.199)	Destabilizing (− 0.83)	Destabilizing (− 0.116)	Destabilizing (− 0.121)
Criteria	PP3_moderate, BS2	BS2	PM2_supporting, BP4	PM2_supporting, PP3_moderate, PS2_moderate	BS2, PP3_moderate, PS2_moderate
Classification	LB	LB	VUS	VUS	VUS

## Discussion

This study identifies a specific cluster of disease-causing missense variants within the SET domain of the *SETD5* gene, which are responsible for an autosomal dominant intellectual developmental disorder, MRD23. While typical cases of MRD23 are caused by LoF variants scattered across the gene, this study suggests that disease-causing missense mutations are concentrated in highly conserved regions essential for the protein’s function.

The *SETD5* gene encodes a protein called SET domain containing 5, which acts as a transcriptional regulator (Dillon et al. 2005). As it contains a conserved SET domain, it is suggested to regulate gene expression by mediating trimethylation of Lys36 of histone H3 (H3K36me3) and through interaction with HDAC3 and PAF1 (Sessa et al. 2019; Deliu et al. 2018; Li et al. 2023). While studies have shown that SETD5 possess intrinsic methyltransferase activity, (Sessa et al. 2019), others claim that it may not function as a canonical methyltransferase, but rather as a scaffold for a co-repressor complex, which includes HDAC3, NCoR, G9a and PAF1 (Li et al. 2023). Either of these essential roles are especially critical in neural progenitors in the developing nervous system, and may drive the observed neurodevelopmental and autistic phenotypes observed in knockout mouse models and in patients with LoF *SETD5* variants.

The first patient with *SETD5* haploinsufficiency was reported by Rauch et al. in 2012 (as part of a non-syndromic ID cohort) (Rauch et al. 2012), and the first *SETD5* patient cohort—defining the MRD23 phenotype—was published by Grozeva et al. (2014). Since then, there have been abundant descriptions of patients with LoF variants or 3p25.3 deletions (n = 104), yet missense variants have been largely overlooked. This is mostly unsurprising, considering that the *SETD5* pLI is 1, while the z score is 2.04 (gnomAD v4.1), suggesting the gene shows constrain towards LoF variants but not towards missense variants. In this context, missense variants appear to be generally unlikely to cause a severe disease. Our current study aimed to define and assess missense variants that emerge as causative of a MRD23-related phenotype, thus providing a framework for diagnosis of these patients. We provide evidence that the region encoding the protein’s SET domain shows high missense constrain (observed/expected 0.2029 for Arg283-Asn390, gnomAD v2.1.1) compared to the rest of the gene (observed/expected > 0.8673 for most of the gene, Fig. 3A, B). By comparing patient data and utilizing computational structural analysis, we distinguish deleterious SET domain variants from likely benign or VUS variants, most of which were located outside the SET domain (Fig. 2, Tables 2 and 3). Structural analysis revealed that likely pathogenic missense variants cluster within two highly conserved and positively charged pockets within the SET domain, one of which is potentially required for H3K36 histone binding (Fig. 2); disease-causing variants affecting residues within these functional pockets likely disrupt the 3D assembly and protein stability necessary for proper transcriptional regulation, driving the MRD23 phenotype. Clinically, in the available data, missense SET domain variant carriers appear to show statistically significant higher rates of autism, hypotonia, dental anomalies and musculoskeletal anomalies, often presenting with more neuropsychiatric and behavioral issues than those with traditional LoF variants of 3p25.3 deletions; these differences described in our series should be interpreted cautiously due to the small sample size of individuals with missense variants, and warrant confirmation in larger harmonized cohorts, rather than aggregated cases from multiple sources as conducted in this study. Notably, our study introduces hydrocephalus as a potentially novel feature associated with this variant type (Table 1), which appeared in two individuals, and expands the known phenotypic spectrum of SETD5-related disorders. Due to lack of sufficient clinical descriptions of individuals with missense variants outside the SET domain, a proper phenotypic comparison was not possible.

Interestingly, we identified a recurring variant (p.Arg348Gln) in three unrelated patients, and another missense variant in the same position (p.Arg348Leu) was previously published as part of the Genomics England cohort (Odhams and Williams 2025). The HPO terms for the patient in the Genomics England cohort were: abnormality of the eye, hypodontia, pectus excavatum, global developmental delay, abnormal facial shape and mild short stature, none of which fit with the disease group this patient was assigned to (renal and urinary tract disorders) and the patient was labeled as undiagnosed; however, his phenotype is in line with MRD23. Importantly, one of the patients (Patient 4) had a methylation signature corresponding to *SETD5*-related disease, which represents functional evidence for its pathogenicity. Taken together, these findings further reinforce these variants’ pathogenicity and identify the Arg348 residue as an apparent pathogenic hotspot for mutagenesis.

Among the observed *SETD5* missense variants, five previously published variants were located outside the SET domain (Table 3): p.Arg77Cys (in a patient with OCD), p.Arg767Cys (in a patient with Moyamoya disease), p.Gly1045Ser (in a patient with a neurodevelopmental phenotype and CAKUT), and p.Lys698Arg and p.Thr727Ala (in patients with neurodevelopmental phenotypes). Unlike disease-causing variants, which are extremely rare, p.Arg767Cys, p.Arg77Cys and p.Thr727Ala were observed in unaffected individuals in gnomAD and the Franklin data analysis software community database (Table 3), and the patient with p.Thr727Ala was also diagnosed with 8p monosomy and partial 4p trisomy. While p.Lys698Arg and p.Gly1045Ser are indeed not found in any databases, their pathogenicity predictions are subpar. This highlights their likely benign or VUS nature compared to the likely pathogenic missense variants in this study, which all localized to the SET domain. It should be noted that not all SET domain variants should be interpreted as de facto pathogenic, and the location of the variant should not be automatically taken as proof of causality; rather, each variant should still be carefully assessed by standard international guidelines.

Defining the SET domain as a critical or hotspot region for pathogenic missense variants refines the understanding of the genetic architecture of MRD23. This would allow more accurate interpretation of *SETD5* variants, and assist in better classification of variants that were previously defined as VUSs. Functional validation of missense variants using an episignature test could provide further proof of variant causality.

In conclusion, we show that *SETD5* missense variants located at the SET domain appear to cause MRD23. Our findings expand the phenotypic features and the mutation spectrum of the *SETD5*-related neurodevelopmental phenotype. Although our study lacks functional assays to prove causality or benign status of each assessed variant, we provide a framework for improving both postnatal and prenatal genetic diagnostics. Further delineation of the clinical spectrum of *SETD5* missense variants is needed to establish these findings.

## Supplementary Information

Below is the link to the electronic supplementary material.

Supplementary file1 (XLSX 14 kb)

## Data Availability

No datasets were generated or analysed during the current study.

## References

[CR1] Abul-Husn NS, Marathe PN, Kelly NR et al (2023) Molecular diagnostic yield of genome sequencing versus targeted gene panel testing in racially and ethnically diverse pediatric patients. Genet Med 25(9):100880. 10.1016/j.gim.2023.100880 PMC10789486 37158195

[CR2] Ahsan N, Desai A, Siddiqui O, Powers K (2023) Expanding the genotype and phenotype of SETD5-related neurodevelopmental syndrome. Pediatr Neurol 138(2023):25–26. 10.1016/j.pediatrneurol.2022.10.001 36335838

[CR3] Ansari M, Halachev M, Parry D et al (2025) Whole genome sequencing of “mutation-negative” individuals with cornelia de lange syndrome. Hum Mutat 2025(1) 10.1155/humu/4711663 PMC12267970 40677927

[CR4] Aoi H, Mizuguchi T, Ceroni JR et al (2019) Comprehensive genetic analysis of 57 families with clinically suspected Cornelia de Lange syndrome. J Hum Genet 64(10):967–978. 10.1038/s10038-019-0643-z 31337854

[CR5] Biesecker LG, Harrison SM (2018) The ACMG/AMP reputable source criteria for the interpretation of sequence variants. Genet Med 20(12):1687–1688. 10.1038/gim.2018.42 PMC6709533 29543229

[CR6] Callahan NC, Mahida S, Sveden A et al (2025) Expansion of the genotypic and phenotypic spectrum of SETD5 disorder using data from the National Brain Gene Registry. Clin Genet 108(3):279–291 10.1111/cge.14746 40265665

[CR7] Chao KR, Wang L, Panchal R et al (2024) The landscape of regional missense mutational intolerance quantified from 125,748 exomes. bioRxiv. 10.1101/2024.04.11.588920v2.abstract

[CR8] ClinGen Sequence Variant Interpretation Group (2020) ClinGen sequence variant interpretation recommendation for PM2—version 1.0 [Internet]. https://clinicalgenome.org/working-groups/sequence-variant-interpretation/

[CR9] ClinGen Sequence Variant Interpretation Group (2021) ClinGen sequence variant interpretation recommendation for de novo criteria (PS2/PM6)—Version 1.1 [Internet]. https://clinicalgenome.org/working-groups/sequence-variant-interpretation/

[CR10] Crippa M, Bestetti I, Maitz S et al (2020) SETD5 gene haploinsufficiency in three patients with suspected KBG syndrome. Front Neurol 11(July):1–9 10.3389/fneur.2020.00631 PMC7393934 32793091

[CR11] Dams L, Milewicz DM, Bauer P, Kraemer M (2026) Moyamoya syndrome in a patient with pathogenic heterozygous variant in the SETD5 gene: a case report. Brain Circ 12(2):183–186 10.4103/bc.bc_78_24 PMC13152241 42111309

[CR12] De Falco A, De Dominicis A, Trivisano M et al (2025) Neurological and psychiatric phenotype of a multicenter cohort of patients with SETD5-related neurodevelopmental disorder. Eur J Paediatr Neurol 54:8–17. 10.1016/j.ejpn.2024.11.008 39603091

[CR13] Deliu E, Arecco N, Morandell J et al (2018) Haploinsufficiency of the intellectual disability gene SETD5 disturbs developmental gene expression and cognition. Nat Neurosci 21(12):1717–1727 10.1038/s41593-018-0266-2 30455454

[CR14] Dillon SC, Zhang X, Trievel RC, Cheng X (2005) The SET-domain protein superfamily: protein lysine methyltransferases. Genome Biol 6(8) 10.1186/gb-2005-6-8-227 PMC1273623 16086857

[CR15] Durkie M, Cassidy E-J, Berry I et al (2024) ACGS best practice guidelines for variant classification in rare disease 2024 [Internet]. http://gnomad.broadinstitute.org/

[CR16] Fan J, Sun H, Jiang H, Zhang S, Xia H, He Y (2025) A case of a fetus with SETD5 mutation: prenatal phenotype and literature review. Birth Defects Res 117(12):1–6 10.1002/bdr2.70003 41368699

[CR17] Fang Y, Zhang R, Wang Y, Cao L, Zhang Y, Cai C (2019) A novel mutation in a common pathogenic gene (SETD5) associated with intellectual disability: a case report. Exp Ther Med:3737–3740 10.3892/etm.2019.8059 PMC6812316 31656537

[CR18] Firth HV, Wright CF (2011) The Deciphering Developmental Disorders (DDD) study. Dev Med Child Neurol 53(8):702–703 10.1111/j.1469-8749.2011.04032.x 21679367

[CR19] Green C, Willoughby J, Balasubramanian M (2017) De novo SETD5 loss-of-function variant as a cause for intellectual disability in a 10-year old boy with an aberrant blind ending bronchus. Am J Med Genet A 173(12):3165–3171 10.1002/ajmg.a.38461 28905509

[CR20] Grozeva D, Carss K, Spasic-Boskovic O et al (2014) De novo loss-of-function mutations in SETD5, encoding a methyltransferase in a 3p25 microdeletion syndrome critical region, cause intellectual disability. Am J Hum Genet 94(4):618–624 10.1016/j.ajhg.2014.03.006 PMC3980521 24680889

[CR21] Kim GJ, Malaquias AC, Bertola DR et al (2025) Non-RASopathy genetic syndromes identified as the molecular cause of disease in patients previously diagnosed with Noonan syndrome. Am J Med Genet A. 10.1002/ajmg.a.64291 41137536

[CR22] Kuechler A, Zink AM, Wieland T et al (2015) Loss-of-function variants of SETD5 cause intellectual disability and the core phenotype of microdeletion 3p25.3 syndrome. Eur J Hum Genet 23(6):753–760 10.1038/ejhg.2014.165 PMC4795044 25138099

[CR23] Li M, Hou Y, Zhang Z et al (2023) Structure, activity and function of the lysine methyltransferase SETD5. Front Endocrinol (Lausanne) 14(February):1–6 10.3389/fendo.2023.1089527 PMC9982096 36875494

[CR24] Lin GN, Song W, Wang W et al (2022) De novo mutations identified by whole-genome sequencing implicate chromatin modifications in obsessive-compulsive disorder. Sci Adv 8(2):1–15 10.1126/sciadv.abi6180 PMC8754407 35020433

[CR25] Luppino G, Wasniewska M, Pepe G et al (2025) Two years of growth hormone therapy in a child with severe short stature due to overlap syndrome with a novel SETD5 gene mutation: case report and review of the literature. Genes (Basel) 16(8):1–10 10.3390/genes16080859 PMC12385900 40869907

[CR26] Manokaran RK, Ochi A, Kerr E et al (2023) Drug-resistant focal epilepsy in a girl with SETD5-related intellectual disability. Seizure 112(2023):109–111. 10.1016/j.seizure.2023.09.025 37797428

[CR27] Odhams C, Williams HJ (2025) Improved diagnosis of patients with rare diseases through the application of constrained coding region annotation and de novo status. Genet Med 27(7):101447. 10.1016/j.gim.2025.101447 40276942

[CR28] Pan M, Liu YN, Xu LL, Li DZ (2020) First-trimester cystic hygroma and neurodevelopmental disorders: the association to remember. Taiwan J Obstet Gynecol 59(6):960–962 10.1016/j.tjog.2020.09.029 33218422

[CR29] Pande S, Majethia P, Nair K et al (2024) De novo variants underlying monogenic syndromes with intellectual disability in a neurodevelopmental cohort from India. Eur J Hum Genet 32(10):1291–1298. 10.1038/s41431-023-01513-7 PMC7616498 38114583

[CR30] Pejaver V, Byrne AB, Feng BJ et al (2022) Calibration of computational tools for missense variant pathogenicity classification and ClinGen recommendations for PP3/BP4 criteria. Am J Hum Genet 109(12):2163–2177 10.1016/j.ajhg.2022.10.013 PMC9748256 36413997

[CR31] Pinard A, Guey S, Guo D et al (2020) The pleiotropy associated with de novo variants in CHD4, CNOT3, and SETD5 extends to moyamoya angiopathy. Genet Med 22(2):427–431 10.1038/s41436-019-0639-2 PMC7673309 31474762

[CR32] Pires SF, Tolezano GC, da Costa SS et al (2020) Expanding the role of SETD5 haploinsufficiency in neurodevelopment and neuroblastoma. Pediatr Blood Cancer 67(11):3–5 10.1002/pbc.28376 32748512

[CR33] Pizzo L, Jensen M, Polyak A et al (2019) Rare variants in the genetic background modulate cognitive and developmental phenotypes in individuals carrying disease-associated variants. Genet Med 21(4):816–825 10.1038/s41436-018-0266-3 PMC6405313 30190612

[CR34] Powis Z, Farwell Hagman KD, Mroske C et al (2018) Expansion and further delineation of the SETD5 phenotype leading to global developmental delay, variable dysmorphic features, and reduced penetrance. Clin Genet 93(4):752–761 10.1111/cge.13132 28881385

[CR35] Rauch A, Wieczorek D, Graf E et al (2012) Range of genetic mutations associated with severe non-syndromic sporadic intellectual disability: an exome sequencing study. Lancet 380(9854):1674–1682 10.1016/S0140-6736(12)61480-9 23020937

[CR36] Richards S, Aziz N, Bale S et al (2015) Standards and guidelines for the interpretation of sequence variants: a joint consensus recommendation of the American College of Medical Genetics and Genomics and the Association for Molecular Pathology. Genet Med 17(5):405–423. 10.1038/gim.2015.30 PMC4544753 25741868

[CR37] Rivera-Munoz EA, Zhao XE, Rosenfeld JA et al (2025) Clinical exome sequencing efficacy and phenotypic expansions involving non-isolated congenital anomalies of kidney and urinary tract (CAKUT+). Eur J Hum Genet 33(12):1606–1615. 10.1038/s41431-025-01929-3 PMC12669671 40913078

[CR38] Sadikovic B, Levy MA, Kerkhof J et al (2021) Clinical epigenomics: genome-wide DNA methylation analysis for the diagnosis of Mendelian disorders. Genet Med 23(6):1065–1074 10.1038/s41436-020-01096-4 PMC8187150 33547396

[CR39] Sessa A, Fagnocchi L, Mastrototaro G et al (2019) SETD5 regulates chromatin methylation state and preserves global transcriptional fidelity during brain development and neuronal wiring. Neuron 104(2):271-289.e13. 10.1016/j.neuron.2019.07.013 31515109

[CR40] Shangguan H, Wang J, Lin J, Huang X, Zeng Y, Chen R (2024) A study on genotypes and phenotypes of short stature caused by epigenetic modification gene variants. Eur J Pediatr 183(3):1403–1414. 10.1007/s00431-023-05385-3 38170291

[CR41] Stur E, Soares LA, Louro ID (2017) SETD5 gene variant associated with mild intellectual disability - a case report. Genet Mol Res 16(2):5–10 10.4238/gmr16029615 28549204

[CR42] Szczałuba K, Brzezinska M, Kot J et al (2016) SETD5 loss-of-function mutation as a likely cause of a familial syndromic intellectual disability with variable phenotypic expression. Am J Med Genet A 170(9):2322–2327 10.1002/ajmg.a.37832 27375234

[CR43] Wright CF, Campbell P, Eberhardt RY et al (2023) Genomic diagnosis of rare pediatric disease in the United Kingdom and Ireland. N Engl J Med 388(17):1559–1571 10.1056/NEJMoa2209046. PMC7614484 37043637

[CR44] Yagasaki H, Toda T, Koizumi K et al (2018) A de novo 10.1-Mb 3p25 terminal deletion including SETD5 in a patient with ptosis and psychomotor retardation. Pediatr Neonatol 59(3):319–321. 10.1016/j.pedneo.2017.09.004 28951171

